# Association of cardiometabolic index with all-cause and cause-specific mortality among overweight and obese adults: a cohort study

**DOI:** 10.3389/fcvm.2025.1610257

**Published:** 2025-06-19

**Authors:** Xi Luo, Bin Cai, Wei-Wei Jin

**Affiliations:** ^1^ Department of Clinical Nutrition, Tongde Hospital of Zhejiang Province, Hangzhou, China; ^2^ Department of Clinical Nutrition, Sir Run Run Shaw Hospital, Zhejiang University School of Medicine, Hangzhou, China; ^3^ School of Medicine, Shaoxing University, Shaoxing, China

**Keywords:** cardiometabolic index, inflammation, overweight, obesity, mortality, national health and nutrition examination survey

## Abstract

**Background:**

This study aimed to explore the associations of cardiometabolic index (CMI) with all-cause and cause-specific mortality among the overweight and obese population.

**Methods:**

Mortality data for 13,674 participants with overweight or obesity were sourced from the National Death Index (NDI) and linked to the National Health and Nutrition Examination Survey (NHANES) datasets. We specifically examined the correlations of CMI with all-cause, premature, and cancer mortality. To ensure a comprehensive analysis, various statistical techniques were employed, including the Cox regression model, subgroup and sensitivity analysis, and restricted cubic spline (RCS) regression analysis. We also explored the potential mediating effect of inflammation-related indicators within these associations.

**Results:**

After adjusting for all covariates, CMI remained positively associated with all-cause, premature, and cancer mortality among overweight and obese adults. For all-cause mortality, the hazard ratio (HR) was 1.14 [95% confidence interval (CI): 1.01–1.28, *P* = 0.041]. For premature mortality, the HR was 1.24 (95% CI: 1.08–1.42, *P* = 0.003). For cancer mortality, the HR was 1.33 (95% CI: 1.08–1.63, *P* = 0.006). When continues CMI was stratified into quartiles, significant correlations were maintained with all-cause mortality (*P* for trend = 0.003), premature mortality (*P* for trend = 0.006), and cancer mortality (*P* for trend = 0.007). Subgroup and sensitivity analyses indicated the robustness of results. Mediation analysis revealed that neutrophils mediated 16.27% of the correlation between CMI and all-cause mortality, and 11.01% of the association between CMI and premature mortality.

**Conclusions:**

Elevated CMI is positively associated with all-cause, premature, and cancer mortality among overweight and obese adults. The associations appeared to be partially mediated by inflammatory pathways, suggesting a mechanism linking CMI to adverse health outcomes. These findings may offer valuable insights for early risk stratification and the formulation of intervention strategies within overweight and obese populations.

## Introduction

Nowadays overweight and obesity have emerged as significant health concerns globally. Numerous studies centered on diverse populations have demonstrated an alarming rise in the number of children, adolescents, and adults affected by overweight or obesity worldwide (1). The association of overweight and obesity with a heightened risk of various non-communicable diseases, such as diabetes mellitus (DM), cardiovascular disease (CVD), and certain cancers, is well-documented. Overweight or obesity not only leads to negative health impacts throughout an individual's life but also contribute to a reduction in life expectancy (1, 2). Consequently, it is imperative to pinpoint modifiable risk factors among those who are overweight or obese to enhance global public health and establish effective preventive strategies.

Chronic low-grade inflammation plays an important role in the onset and progression of obesity (3). Earlier research has demonstrated that excessive body fat accumulation can induce an imbalanced production of various adipokines and promote the infiltration of macrophages and other immune cells in adipose tissue (AT) (4, 5). Elevated inflammatory markers are independently associated with increased tissue damage and mortality risks in obese adults, irrespective of other established risk factors (6, 7). Therefore, systemic inflammation might considerably affect the long-term prognosis of individuals within the obese population.

The cardiometabolic index (CMI) is a metabolism-related indicator that combines the weight-to-height ratio (WHtR) with biochemical lipid parameters. It was initially developed as an indicator to predict the risk of DM (8). Subsequent studies have further explored the correlation between CMI and DM among diverse populations from regions including China, Japan, and the United States (U.S.) (9–11). Additionally, extensive research has demonstrated that CMI is positively correlated with increased risks of chronic conditions such as hypertension, CVD, and metabolic syndrome (MetS) (12–15). As CMI's application in clinical settings has grown, research has also highlighted its prognostic significance for all-cause mortality and mortality related to CVD and cancer across both elderly and general populations (16–18). Nonetheless, there is a notable gap in previous research regarding the relationship between CMI with all-cause, premature, and cancer mortality specifically among overweight and obese adults.

Therefore, the aim of the present study was to explore the associations of CMI with all-cause and cause-specific mortality in overweight and obese adults in the U.S. using the data from the National Health and Nutrition Examination Survey (NHANES) from 1999 to 2018. Furthermore, we investigated the potential role of inflammatory indicators in mediating the link between CMI and mortality.

## Materials and methods

### Subject population and design

**Figure 1:**
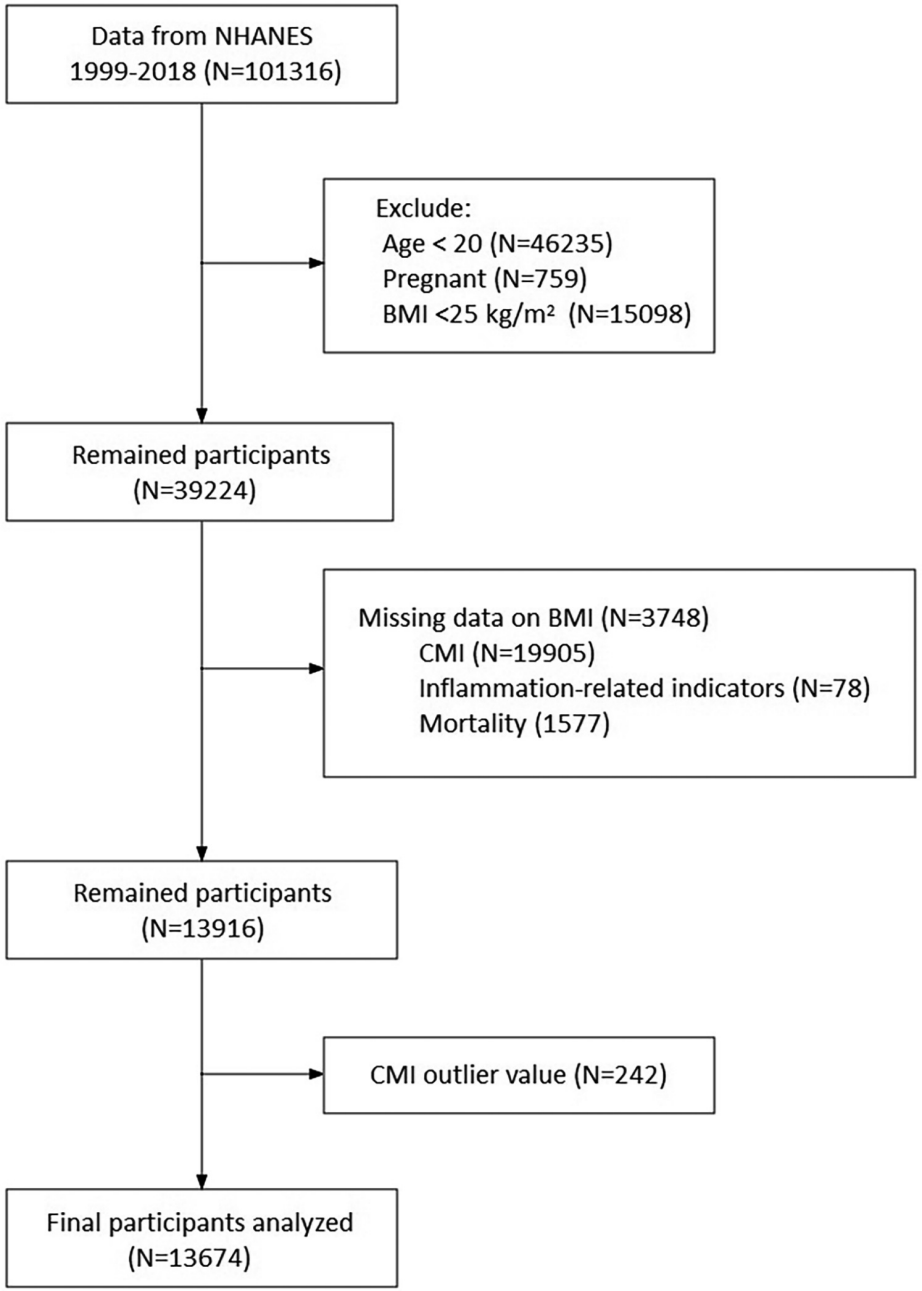
Flow chart of study participants. NHANES, National Health and Nutrition Examination Survey; CMI, cardiometabolic index; BMI, body mass index.

This study is a longitudinal cohort analysis based on data obtained from the NHANES. Comprehensive details about the survey methodologies and data access can be found at https://www.cdc.gov/nchs/nhanes/about_nhanes.htm. We extracted an extensive array of data from the NHANES database, including demographic information, questionnaire responses, physical examination results, and health-related data, spanning the years 1999–2018. To uphold the integrity and reliability of results, our exclusion criteria included individuals <20 years of age, pregnant, body mass index (BMI) < 25.0 kg/m^2^, missing data on BMI, CMI, inflammation-related indicators, mortality, and CMI outlier value. Finally, a total of 13,674 participants were enrolled in the present study (Figure 1).

### Exposure variable and outcomes

The exposure variable was CMI. CMI = [Triglyceride (TG, mmol/L)/high-density lipoprotein cholesterol (HDL-c, mmol/L)] × [waist circumference (WC, cm)/height (cm)] (8).

The outcome variables were all-cause, premature, cancer, DM, and cardiovascular mortality. Participant mortality data were obtained from the National Center for Health Statistics (NCHS). The NHANES use linked mortality file with National Death Index (NDI) from NCHS using a well-documented and validated method of matching deaths to population data sets. Mortality follow-up data were available from the most recent update of December 31, 2019. The cause of death was determined using codes of International Statistical Classification of Diseases (10th Revision, ICD-10). Cancer mortality referred to malignant neoplasms mortality and identified through codes ranging from C00 to C97. DM mortality was identified through codes ranging from E10 to E14. Cardiovascular mortality was identified through codes of I00-I09, I11, I13, I20-I51. Premature mortality was assessed using permth_int, and defined as death occurring before the age of 75 (19).

### Assessment of inflammatory indicators

Following the NHANES cohort, laboratory parameters including lymphocytes, neutrophils, monocytes, platelets were enrolled. The complete blood count (CBC)-derived systemic inflammatory indicators, including systemic immune-inflammation index (SII), aggregate index of systemic inflammation (AISI), and systemic inflammation response index (SIRI) were calculated by following formulas:

SII = (platelet count × neutrophil count)/lymphocyte count (16).

AISI = (neutrophil count × platelet count × monocyte count)/lymphocyte count (20).

SIRI = (neutrophil count × monocyte count)/lymphocyte count (21).

### Covariates

We obtained the particular methodologies and caliber of determination for every covariate control approach from NHANES (https://www.cdc.gov/nchs/nhanes/about_nhanes.htm). The covariates of present study including age, gender, race, marital status, education level, family poverty-to-income ratio (PIR), smoking status, drinking status, DM, hypertension and hyperlipidemia history. DM was diagnosed based on self-reported history of DM, use of insulin or oral antidiabetic medications, fasting glucose ≥7.0 mmol/L, two-hour glucose (OGTT) ≥11.1 mmol/L, or glycosylated hemoglobin (HbAlc) ≥6.5%. The presence of hypertension was suggested by one of the following: self-reported history of hypertension, told had high blood pressure 2+ times, taking prescription for hypertension, systolic blood pressure (BP) ≥140 mmHg, or diastolic BP ≥90 mmHg. Hyperlipidemia was identified in participants who were taking lipid-lowering medications, TG ≥1.7 mmol/L, total cholesterol (TC) ≥ 5.2 mmol/L, low-density lipoprotein cholesterol (LDL-C) ≥ 3.4 mmol/L, and/or HDL-C ≤ 1.0 mmol/L for males or ≤1.3 mmol/L for females (22).

### Statistical analysis

All analyses were conducted using R version 4.3.0 (The R Foundation, http://www.R-project.org), along with the Zstats v0.90 (http://www.medsta.cn/software), and EmpowerStats (http://www.empower stats.net, X&Y Solutions, Inc., Boston, Massachusetts). A *P*-value of <0.05 was considered statistically significant. Given the complex multistage cluster survey design of the NHANES study, we utilized sample weighting codes “WTSAF4YR” and “WTSAF2YR” for the fasting subsample from 1999 to 2002 and 2003 to 2018, respectively.

To describe the baseline characteristics, continuous variables were expressed as mean (standard error, SE), while categorical variables were presented as number (*N*) (percentages, %). We compared the baseline characteristics across CMI quartiles, and the cut-offs of CMI are 0.68, 0.54, 0.80 and 1.13.

In the first part of present study, we investigated the associations of CMI with all-cause and cause-specific mortality among the overweight and obese adults based on datasets from NHANES. Our statistical analyses consisted of 3 main steps. First, we constructed Cox regression analysis with univariate and multivariate models. Model 1 was non-adjusted model. Model 2 was modified for age, gender, race, marital status, education level, PIR, smoking status, and drinking status. Model 3 was further modified for comorbidities including DM, hypertension and hyperlipidemia history were adjusted. Second, we constructed the restricted cubic spline (RCS) analysis for CMI with all-cause, premature, and cancer mortality with multivariate model (adjust for age, gender, race, marital status, education level, PIR, smoking status, drinking status, DM, hypertension and hyperlipidemia). Third, to strengthen the reliability of our data analysis, we implemented both sensitivity and subgroup analyses. Initially, to address missing values in variables such as marital status (1.04%), education level (0.06%), PIR (8.32%), smoking status (0.10%), drinking status (16.28%), DM (1.29%), and hypertension (0.30%), we employed multiple imputation techniques. This process involved five replications and utilized the Markov-chain Monte Carlo method within the SAS MI procedure. RCS analysis was applied before and after imputation. Subsequently, we performed stratified and interaction analyses across various parameters, including age, gender, BMI, race, marital status, education level, smoking status, drinking status, and histories of DM, hypertension, and hyperlipidemia.

In the second part of present study, we investigated the mediation by inflammatory indicators of the association between CMI and mortality.

## Results

### Baseline characteristics of study population

The present study extracted data from the NHANES database spanning the years 1999–2018. Individuals were exclude based on the following criteria: age <20 years (*N* = 46,235), pregnancy (*N* = 759), body mass index (BMI) < 25.0 kg/m^2^ (*N* = 15,098), missing data on BMI (*N* = 3,748), missing data on CMI (*N* = 19,905), missing data on inflammation-related indicators (*N* = 78), missing data on mortality (*N* = 1,577), and CMI outlier value (*N* = 242). After exclusions, a total of 13,674 participants were enrolled in the present study (Figure 1). The weighted distribution of selected participant characteristics according to CMI quartiles is shown in Table 1. Significant differences were observed between CMI quartiles for all included baseline characteristics. Compared to Quartile 1 of CMI, participants in Quartile 4 were more likely to be older, male, Mexican American or Non-Hispanic White, have a lower education level (below high school), be married, be current smokers, and have a higher prevalence of DM, hypertension, or hyperlipidemia history, as well as higher levels of BMI, WC, WHtR, TG/HDL-c, and inflammation indicators (all *P* < 0.001). The mortality rates for all-cause, premature, cancer, DM, and cardiovascular were significantly higher in Quartile 4 (all *P*-values <0.01), highlighting the importance of exploring the associations of CMI with all-cause and cause-specific mortality. We also explored the differences of CMI, inflammation, and mortality in different obesity class groups (overweight, Class I, II, III obesity) (Supplementary Table 1).

**Table 1:** Characteristics of study population.

Characteristic	CMI				Statistic	*P* value
	Q 1	Q 2	Q 3	Q 4		
N^a^	3,542	3,494	3,393	3,245		
Gender, Male, *N* (%)	1,382 (41.24)	1,677 (48.88)	1,779 (56.05)	1,937 (61.69)	*χ*^2^ = 322.88	<0.001
Age, years, Mean (SE)	44.07 (0.46)	45.71 (0.36)	47.06 (0.31)	46.92 (0.33)	*F* = 34.44	<0.001
Race, *N* (%)					*χ*^2^ = 391.97	<0.001
Mexican American	520 (7.80)	748 (9.90)	809 (10.09)	844 (10.61)		
Other Hispanic	313 (6.21)	339 (6.52)	361 (7.23)	312 (6.01)		
Non-Hispanic White	1,212 (61.49)	1,324 (65.94)	1,400 (68.22)	1,523 (72.49)		
Non-Hispanic Black	1,290 (20.08)	843 (12.74)	573 (8.73)	349 (5.77)		
Other race—including multi-racial	207 (4.41)	240 (4.91)	250 (5.73)	217 (5.12)		
Education, *N* (%)					*χ*^2^ = 159.99	<0.001
Less than 9th grade	282 (4.59)	406 (6.17)	475 (7.12)	515 (7.37)		
9–11th grade	449 (9.22)	533 (11.21)	533 (13.06)	553 (13.79)		
High School or Equivalent	762 (22.76)	812 (24.38)	815 (26.55)	777 (26.85)		
Some College or AA degree	1,155 (32.66)	1,032 (32.54)	926 (29.22)	900 (31.54)		
College Graduate or above	893 (30.76)	709 (25.70)	642 (24.04)	497 (20.45)		
Marital status, *N* (%)					*χ*^2^ = 76.39	<0.001
Married	1,751 (56.23)	1,922 (59.23)	1,963 (62.66)	1,886 (62.41)		
Widowed	155 (3.24)	164 (3.19)	188 (4.13)	169 (3.99)		
Divorced	405 (10.95)	396 (11.22)	343 (9.26)	370 (10.76)		
Separated	139 (2.70)	142 (2.88)	119 (2.79)	103 (2.18)		
Never married	737 (18.76)	558 (15.57)	472 (14.05)	420 (13.15)		
Living with partner	328 (8.13)	284 (7.92)	261 (7.11)	257 (7.51)		
Smoking status, yes, *N* (%)	1,328 (39.52)	1,535 (45.94)	1,625 (50.41)	1,744 (53.92)	*χ*^2^ = 158.46	<0.001
Alcohol status, yes, *N* (%)	2,034 (77.14)	2,097 (75.82)	2,039 (74.95)	2,017 (74.60)	*χ*^2^ = 5.86	0.319
PIR, Mean (SE)	3.17 (0.05)	3.05 (0.04)	2.97 (0.05)	2.94 (0.05)	*F* = 21.01	<0.001
BMI, kg/m^2^, Mean (SE)	29.77 (0.11)	31.01 (0.12)	32.41 (0.14)	33.51 (0.18)	*F* = 337.56	<0.001
Waist circumference, cm, Mean (SE)	98.68 (0.26)	103.21 (0.31)	107.74 (0.35)	111.34 (0.42)	*F* = 631.97	<0.001
WHtR, Mean (SE)	0.59 (0.00)	0.61 (0.00)	0.64 (0.00)	0.65 (0.00)	*F* = 493.16	<0.001
TG/HDL-c, Mean (SE)	0.48 (0.00)	0.88 (0.00)	1.40 (0.01)	2.76 (0.02)	*F* = 9,193.96	<0.001
CMI, Mean (SE)	0.28 (0.00)	0.53 (0.00)	0.88 (0.00)	1.78 (0.01)	*F* = 9,704.00	<0.001
Lymphocytes, 1,000 cells/ul, Mean (SE)	1.88 (0.01)	2.02 (0.02)	2.08 (0.01)	2.17 (0.02)	*F* = 234.06	<0.001
Neutrophils, 1,000 cell/ul, Mean (SE)	3.67 (0.04)	3.99 (0.03)	4.22 (0.03)	4.47 (0.04)	*F* = 298.78	<0.001
Monocytes, 1,000 cells/ul, Mean (SE)	0.51 (0.00)	0.54 (0.00)	0.56 (0.00)	0.58 (0.01)	*F* = 124.09	<0.001
Platelets, 1,000 cells/ul, Mean (SE)	248.92 (1.58)	256.30 (1.66)	258.97 (1.69)	259.78 (1.52)	*F* = 27.99	<0.001
SII, Mean (SE)	526.90 (8.18)	544.63 (6.10)	565.61 (7.83)	575.57 (6.97)	*F* = 9.38	<0.001
AISI, Mean (SE)	280.67 (6.05)	300.42 (4.63)	320.32 (5.36)	339.77 (6.03)	*F* = 19.30	<0.001
SIRI, Mean (SE)	1.11 (0.02)	1.16 (0.02)	1.23 (0.02)	1.29 (0.02)	*F* = 16.60	<0.001
DM, yes, *N* (%)	419 (7.47)	633 (12.55)	815 (17.95)	995 (23.87)	*χ*^2^ = 385.55	<0.001
Hypertension, yes, *N* (%)	1,303 (30.95)	1,475 (36.87)	1,638 (44.98)	1,617 (47.98)	*χ*^2^ = 255.35	<0.001
Hyperlipidemia, yes, *N* (%)	1,856 (52.08)	2,539 (73.94)	3,073 (90.27)	3,239 (99.75)	*χ*^2^ = 2,696.46	<0.001
All-cause mortality, yes, *N* (%)	209 (4.89)	315 (7.98)	389 (10.43)	427 (13.26)	*χ*^2^ = 156.57	<0.001
Premature mortality, yes, *N* (%)	135 (3.21)	191 (4.82)	230 (6.56)	283 (9.70)	*χ*^2^ = 138.81	<0.001
Cancer mortality, yes, *N* (%)	50 (1.26)	92 (2.24)	102 (2.68)	120 (3.91)	*χ*^2^ = 50.67	<0.001
DM mortality, yes, *N* (%)	7 (0.18)	10 (0.21)	19 (0.31)	34 (0.68)	*χ*^2^ = 15.88	0.006
Cardiovascular mortality, yes, *N* (%)	52 (1.21)	78 (2.03)	104 (2.71)	108 (3.39)	*χ*^2^ = 39.36	<0.001
Follow-up time, months, Mean (SE)	123.84 (2.08)	130.77 (1.93)	139.57 (2.17)	141.74 (2.18)	*F* = 61.59	<0.001

Mean (SE) for continuous variables. *N* (%) for categorical variables. The quintile cut-off values of the CMI are 0.68, 0.54, 0.80 and 1.13. Q, quartile; N, number SE, standard error; CMI, cardiometabolic index; PIR, family poverty-to-income ratio; BMI, body mass index; WHtR, waist to height ratio; TG, triglyceride; HDL-c, high-density lipoprotein cholesterol; SII, systemic immune-inflammation index; AISI, aggregate index of systemic inflammation; SIRI, systemic inflammation response index; DM, diabetes mellitus.

^a^ Unweighted number of observations in dataset.

### Associations of CMI with all-cause and cause-specific mortality

**Figure 2:**
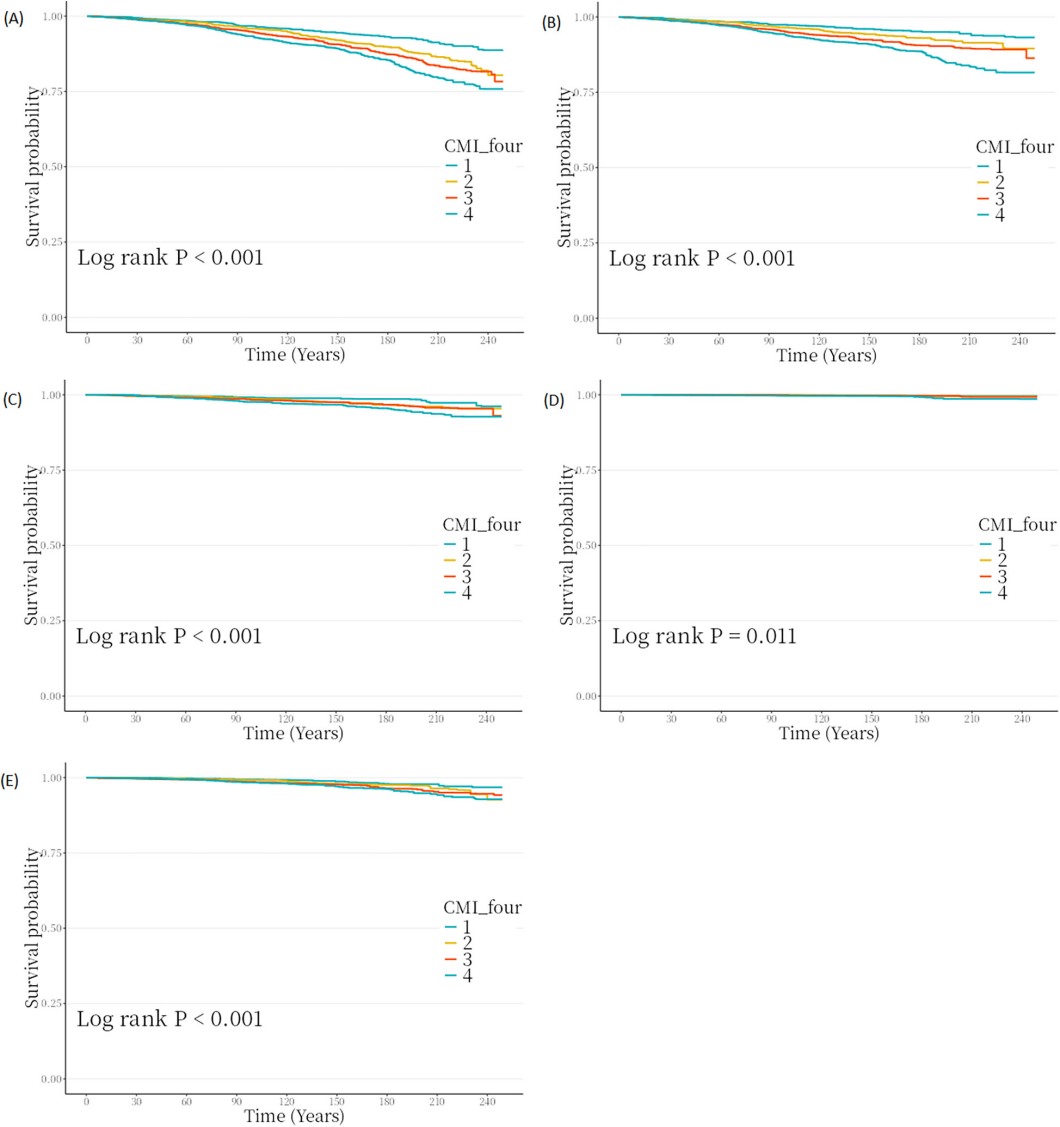
Kaplan–meier survival analysis curves for **(A)** all-cause, **(B)** premature, and **(C)** cancer, **(D)** DM, and **(E)** cardiovascular mortality.

The median follow-up duration was 132.00 months (IQR: 70.00–205.00 months). First, we performed the Kaplan–Meier survival analysis with unadjusted model, which showed that individuals in the Quartile 4 had the least follow-up time with statistical significance (all *P*-values <0.05, Figure 2). Then, we constructed Cox regression analysis with univariate and multivariate models for analyzing the independent role of CMI in all-cause and cause-specific mortality. The HRs and 95% CIs for crude and adjusted models are shown in Table 2. In the crude Model (Model 1, Table 2), continuous CMI showed significant associations with all-cause (HR = 2.09, 95% CI: 1.35–3.24, *P* = 0.001), premature (HR = 1.53, 95% CI: 1.36–1.73, *P* < 0.001), cancer (HR= 1.44, 95% CI: 1.19–1.73, *P* < 0.001), DM (HR = 2.09, 95% CI: 1.51–2.90, *P* < 0.001), and cardiovascular mortality (HR = 2.09, 95% CI: 1.51–2.90, *P* < 0.001). In the fully adjusted model (Model 3, Table 2), significant associations sustained positive and significant in all-cause mortality (HR = 1.14, 95% CI: 1.01–1.28, *P* = 0.041), premature mortality (HR = 1.24, 95% CI: 1.08–1.42, *P* = 0.003), and cancer mortality (HR = 1.33, 95% CI: 1.08–1.63, *P* = 0.006). We also converted CMI into quartiles, and categorical CMI displayed significant correlations with all-cause (*P* for trend <0.001), premature (*P* for trend <0.001), cancer (*P* for trend <0.001), DM (*P* for trend = 0.021) and cardiovascular mortality (*P* for trend <0.001) in the crude Model (Model 1, Table 2). After completely adjustment (Model 3, Table 2), categorical CMI still existed significant correlations with all-cause (*P* for trend = 0.003), premature (*P* for trend = 0.006), and cancer (*P* for trend = 0.006). Additionally, we also assessed the correlations of BMI with mortality (Supplementary Table 2).

**Table 2:** The associations of CMI with all-cause mortality and cause-specific mortality.

Characteristic	Model 1	Model 2	Model 3
	HR (95%CI), *P* value	HR (95%CI), *P* value	HR (95%CI), *P* value
All-cause mortality			
CMI per unit increase	2.09 (1.35, 3.24), 0.001	1.20 (1.06, 1.35), 0.003	1.14 (1.01, 1.28), 0.041
CMI quartile			
Q 1	Ref.	Ref.	Ref.
Q 2	1.53 (1.21, 1.92), <0.001	1.28 (1.01, 1.63), 0.046	1.24 (0.97, 1.58), 0.088
Q 3	1.81 (1.43, 2.30), <0.001	1.29 (1.03, 1.62), 0.025	1.15 (1.01, 1.55), 0.046
Q 4	2.27 (1.78, 2.90), <0.001	1.64 (1.26, 2.13), <0.001	1.56 (1.19, 2.04), 0.001
*P* for trend	<0.001	<0.001	0.003
Premature mortality			
CMI per unit increase	1.53 (1.36, 1.73), <0.001	1.34 (1.16, 1.53), <0.001	1.24 (1.08, 1.42), 0.003
CMI quartile			
Q 1	Ref.	Ref.	Ref.
Q 2	1.44 (1.06, 1.97), 0.021	1.36 (0.97, 1.89), 0.074	1.36 (0.99, 1.87), 0.059
Q 3	1.84 (1.38, 2.45), <0.001	1.37 (1.00, 1.89), 0.052	1.32 (0.95, 1.83), 0.100
Q 4	2.65 (2.07, 3.40), <0.001	2.01 (1.47, 2.75), <0.001	1.86 (1.28, 2.69), 0.001
*P* for trend	<0.001	<0.001	0.006
Cancer mortality			
CMI per unit increase	1.44 (1.19, 1.73), <0.001	1.30 (1.06, 1.60), 0.012	1.33 (1.08, 1.63), 0.006
CMI quartile			
Q 1	Ref.	Ref	Ref.
Q 2	1.67 (0.99, 2.84), 0.056	1.47 (0.82, 2.62), 0.193	1.55 (0.82, 2.93), 0.180
Q 3	1.83 (1.16, 2.88), 0.009	1.44 (0.88, 2.34), 0.143	1.55 (0.89, 2.71), 0.124
Q 4	2.63 (1.57, 4.41), <0.001	2.18 (1.23, 3.87), 0.008	2.44 (1.29, 4.59), 0.006
*P* for trend	<0.001	0.013	0.007
DM mortality			
CMI per unit increase	2.09 (1.51, 2.90), <0.001	2.00 (1.27, 3.15), 0.003	1.42 (0.89, 2.29), 0.143
CMI quartile			
Q 1	Ref.	Ref.	Ref.
Q 2	1.11 (0.28, 4.48), 0.882	1.55 (0.28, 8.65), 0.620	1.07 (0.17, 6.90), 0.994
Q 3	1.43 (0.42, 4.86), 0.562	1.80 (0.38, 8.57), 0.462	1.01 (0.16, 6.23), 0.992
Q 4	3.13 (0.95, 10.36), 0.061	3.47 (0.70, 17.21), 0.128	1.48 (0.21, 10.33), 0.695
*P* for trend	0.021	0.056	0.570
Cardiovascular mortality			
CMI per unit increase	2.09 (1.51, 2.90), <0.001	1.24 (0.99, 1.56), 0.058	1.16 (0.94, 1.43), 0.158
CMI quartile			
Q 1	Ref.	Ref.	Ref.
Q 2	1.57 (0.97, 2.54), 0.069	1.33 (0.80, 2.22), 0.275	1.22 (0.71, 2.07), 0.472
Q 3	1.89 (1.22, 2.91), 0.004	1.27 (0.81, 2.01), 0.296	1.18 (0.75, 1.86), 0.469
Q 4	2.32 (1.51, 3.58), <0.001	1.63 (0.98, 2.69), 0.059	1.46 (0.86, 2.48), 0.161
*P* for trend	<0.001	0.094	0.161

The quintile cut-off values of the CMI are 0.68, 0.54, 0.80 and 1.13. Model 1: No covariates were adjusted. Model 2: age, gender, race, marital status, education level, PIR, smoking status, and drinking status. Model 3: age, gender, race, marital status, education level, PIR, smoking status, drinking status, DM, hypertension, and hyperlipidemia were adjusted. Q, quartile; HR, hazard ratio; 95% CI, 95% confidence interval; CMI, cardiometabolic index; PIR, family poverty-to-income ratio; DM, diabetes mellitus.

### Non-linear relationships

**Figure 3:**
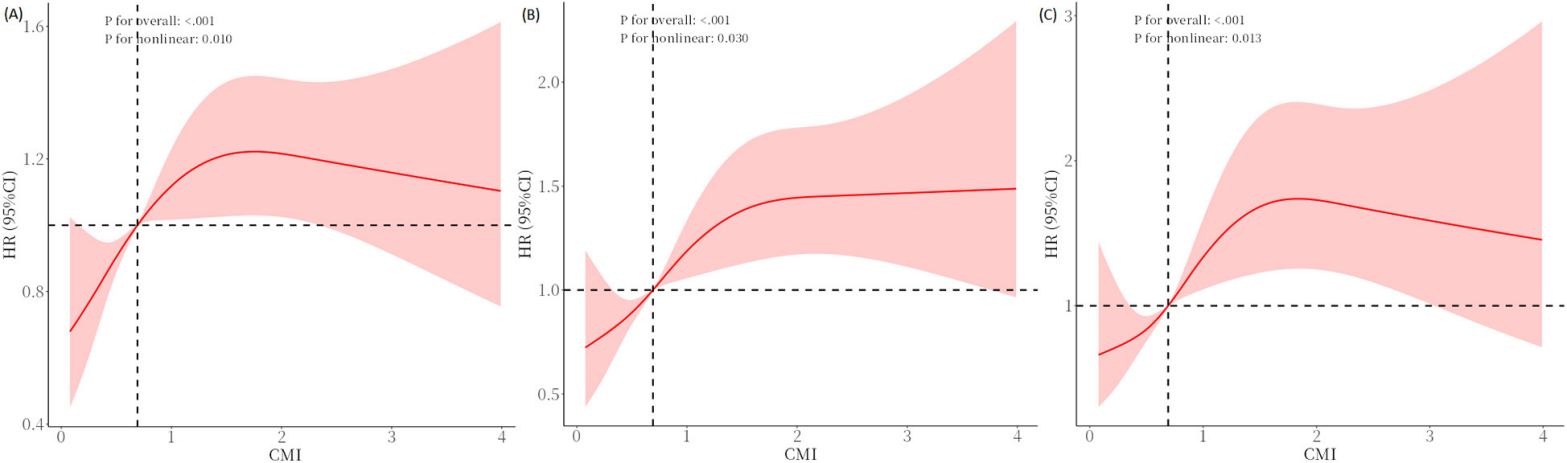
RCS regression analysis for CMI with **(A)** all-cause, **(B)** premature, and **(C)** cancer mortality. Adjust for age, gender, race, marital status, education level, PIR, smoking status, drinking status, DM, hypertension and hyperlipidemia. Adjust for age, gender, race, marital status, education level, PIR, smoking status, drinking status, DM, hypertension and hyperlipidemia. RCS, restricted cubic spline; CMI, cardiometabolic index; PIR, family poverty-to-income ratio; DM, diabetes mellitus.

We further explored the associations between CMI and all-cause, premature, and cancer mortality by employing RCS analysis. As demonstrated in Figure 3, there was an inverted L-shaped non-linear correlation between CMI and all-cause (*P* for overall <0.001; *P* for non-linearity = 0.010), premature (*P* for overall <0.001; *P* for non-linearity = 0.030), and cancer mortality (*P* for overall <0.001; *P* for non-linearity = 0.013).

### Subgroup and sensitivity analyses

To ensure the robustness of our findings, we reexamined the associations of CMI with all-cause, premature, and cancer mortality using imputed data. The analysis confirmed that the findings were consistent with those from the main analyses, as detailed in Supplementary Figure 1. Then, we used age, gender, BMI, race, education level, marital status, smoking status, drinking status, DM, hypertension, and hyperlipidemia history as stratification variables and performed stratified analyses to evaluate the associations of CMI with all-cause, premature, and cancer mortality in stratified populations. Supplementary Table 1 demonstrated that the associations of CMI with all-cause, premature, and cancer mortality generally remained stable. Positive correlations (*P* for interaction <0.05) were observed in strata defined by age, race, marital status, smoking status, and hyperlipidemia. However, positive correlations of CMI with all-cause, premature, or cancer mortality consistently persisted across these groups (Supplementary Table 3).

### Associations of inflammatory indicators with CMI and mortality

Table 3 demonstrated the correlations of CMI with inflammatory indicators by univariate and multivariate linear regression. After adjusting all covariates (Model 3: age, gender, race, marital status, education level, PIR, smoking status, and drinking status, DM, hypertension and hyperlipidemia), CMI still was positively associated with lymphocytes (*β* = 0.15, 95% CI = 0.12–0.18, *P* < 0.001), neutrophils (*β* = 0.32, 95% CI = 0.24–0.39, *P* < 0.001), monocytes (*β* = 0.02, 95% CI = 0.01–0.03, *P* < 0.001), platelets (*β* = 7.40, 95% CI = 4.40–10.41, *P* < 0.001), SII (*β* = 14.59, 95% CI = 0.98–28.20, *P* = 0.038), AISI (*β* = 17.87, 95% CI = 5.45–30.30, *P* = 0.006), and SIRI (*β* = 0.04, 95% CI = 0.01–0.09, *P* = 0.045). Cox regression models of inflammatory indicators with all-cause, premature, and cancer mortality are shown in Table 4. Most indicators were significantly positively related to mortality, except lymphocytes with all-cause and premature mortality, platelets with all-cause, premature, and cancer mortality, monocytes with cancer mortality, and SIRI with cancer mortality.

**Table 3:** The associations between CMI and inflammation-related indicators.

Characteristic	Model 1		Model 2		Model 3	
	*β* (95%CI)	*P*	*β* (95%CI)	*P*	*β* (95%CI)	*P*
Lymphocytes	0.16 (0.13, 0.18)	<0.001	0.18 (0.15. 0.21)	<0.001	0.15 (0.12, 0.18)	<0.001
Neutrophils	0.42 (0.37, 0.47)	<0.001	0.38 (0.31, 0.45)	<0.001	0.32 (0.24, 0.39)	<0.001
Monocytes	0.03 (0.02, 0.04)	<0.001	0.02 (0.01, 0.03)	<0.001	0.02 (0.01, 0.03)	<0.001
Platelets	4.19 (1.97, 6.42)	<0.001	9.34 (6.62, 12.07)	<0.001	7.40 (4.40, 10.41)	<0.001
SII	23.20 (13.33, 33.07)	<0.001	20.57 (7.90, 33.25)	0.002	14.59 (0.98, 28.20)	0.038
AISI	28.45 (19.84, 37.06)	<0.001	23.06 (12.01, 34.11)	<0.001	17.87 (5.45, 30.30)	0.006
SIRI	0.09 (0.06, 0.12)	<0.001	0.05 (0.01, 0.09)	0.014	0.04 (0.01, 0.09)	0.045

Model 1: no covariates were adjusted. Model 2: age, gender, race, marital status, education level, PIR, smoking status, and drinking status. Model 3: age, gender, race, marital status, education level, PIR, smoking status, drinking status, DM, hypertension, and hyperlipidemia were adjusted. HR, hazard ratio; 95% CI, 95% confidence interval; CMI, cardiometabolic index; SII, systemic immune-inflammation index; AISI, aggregate index of systemic inflammation; SIRI, systemic inflammation response index; PIR, family poverty-to-income ratio; DM, diabetes mellitus.

**Table 4:** The associations of inflammation-related indicators with mortality.

Characteristic	Model 1		Model 2		Model 3	
	HR (95%CI)	*P*	HR (95%CI)	*P*	HR (95%CI)	*P*
All-cause mortality						
Lymphocytes	0.94 (0.80, 1.10)	0.453	1.02 (0.96, 1.09)	0.488	1.02 (0.94, 1.10)	0.624
Neutrophils	1.10 (1.07, 1.12)	<0.001	1.09 (1.07, 1.12)	<0.001	1.08 (1.06, 1.11)	<0.001
Monocytes	2.67 (1.89, 3.79)	<0.001	2.05 (1.47, 2.87)	<0.001	1.92 (1.40, 2.65)	<0.001
Platelets	0.99 (0.99, 0.99)	<0.001	1.00 (1.00, 1.00)	0.689	1.00 (1.00, 1.00)	0.898
SII	1.01 (1.01, 1.01)	<0.001	1.01 (1.01, 1.01)	<0.001	1.01 (1.01, 1.01)	<0.001
AISI	1.01 (1.01, 1.01)	<0.001	1.01 (1.01, 1.01)	<0.001	1.01 (1.01, 1.01)	<0.001
SIRI	1.30 (1.23, 1.37)	<0.001	1.27 (1.21, 1.34)	<0.001	1.24 (1.18, 1.31)	<0.001
Premature mortality						
Lymphocytes	0.95 (0.78, 1.16)	0.612	0.97 (0.85, 1.12)	0.716	0.94 (0.80, 1.09)	0.411
Neutrophils	1.10 (1.08, 1.13)	<0.001	1.09 (1.07, 1.12)	<0.001	1.08 (1.04, 1.11)	<0.001
Monocytes	2.72 (1.86, 3.98)	<0.001	1.90 (1.29, 2.78)	0.001	1.70 (1.16, 2.49)	0.006
Platelets	0.99 (0.99, 0.99)	0.025	1.00 (1.00, 1.00)	0.494	1.00 (1.00, 1.00)	0.619
SII	1.01 (1.01, 1.01)	<0.001	1.01 (1.01, 1.01)	<0.001	1.01 (1.01, 1.01)	<0.001
AISI	1.01 (1.01, 1.01)	<0.001	1.01 (1.01, 1.01)	<0.001	1.01 (1.01, 1.01)	<0.001
SIRI	1.32 (1.25, 1.39)	<0.001	1.29 (1.22, 1.36)	<0.001	1.24 (1.17, 1.32)	<0.001
Cancer mortality						
Lymphocytes	1.11 (1.01, 1.23)	0.046	1.12 (1.05, 1.20)	<0.001	1.12 (1.05, 1.20)	<0.001
Neutrophils	1.09 (1.05, 1.13)	<0.001	1.07 (1.01, 1.15)	0.037	1.08 (1.01, 1.15)	0.035
Monocytes	2.36 (1.50, 3.71)	<0.001	1.56 (0.82, 2.99)	0.177	1.53 (0.80, 2.95)	0.202
Platelets	0.99 (0.99, 0.99)	0.040	1.00 (1.00, 1.00)	0.552	1.00 (1.00, 1.00)	0.694
SII	1.01 (1.01, 1.01)	<0.001	1.00 (1.00, 1.00)	0.138	1.00 (1.00, 1.00)	0.050
AISI	1.01 (1.01, 1.01)	<0.001	1.01 (1.01, 1.01)	0.002	1.01 (1.01, 1.01)	0.001
SIRI	1.23 (1.14, 1.33)	<0.001	1.12 (0.96, 1.30)	0.152	1.11 (0.95, 1.31)	0.193

Model 1: no covariates were adjusted. Model 2: age, gender, race, marital status, education level, PIR, smoking status, and drinking status. Model 3: age, gender, race, marital status, education level, PIR, smoking status, drinking status, DM, hypertension, and hyperlipidemia were adjusted. HR, hazard ratio; 95% CI, 95% confidence interval; SII, systemic immune-inflammation index; AISI, aggregate index of systemic inflammation; SIRI, systemic inflammation response index; PIR, family poverty-to-income ratio; DM, diabetes mellitus.

### Mediating role of inflammatory indicators

**Figure 4:**
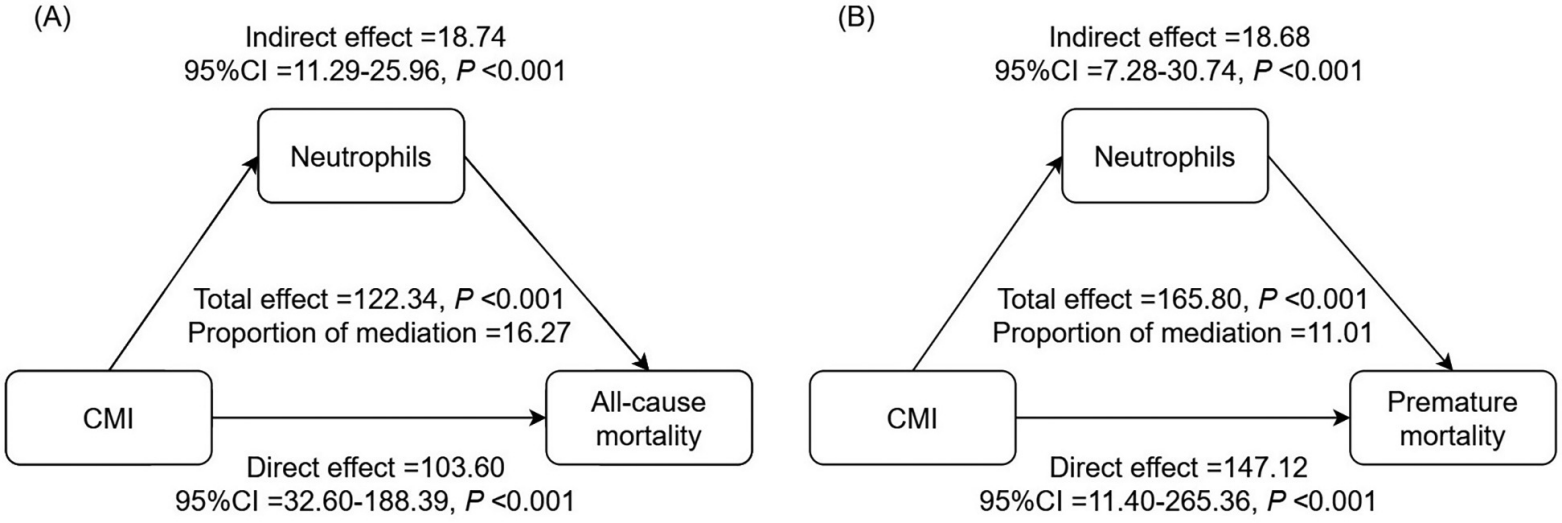
Analysis of the mediation by neutrophils of the associations of CMI with **(A)** all-cause, and **(B)** premature mortality. Adjust for age, gender, race, marital status, education level, PIR, smoking status, drinking status, DM, hypertension and hyperlipidemia. RCS, restricted cubic spline; CMI, cardiometabolic index; PIR, family poverty-to-income ratio; DM, diabetes mellitus.

Mediation analysis revealed that neutrophils mediated 16.27% of the correlation between CMI and all-cause mortality, and 11.01% of the association between CMI and premature mortality (Figure 4). Additionally, we also assessed the mediating roles of other inflammatory indicators including lymphocytes, neutrophils, monocytes, platelets, SII, AISI, and SIRI (Supplementary Table 4).

## Discussion

The present study is the first to explore the associations of the CMI with all-cause, premature, and cancer mortality among overweight and obese adults in the U.S., using a substantial prospective cohort. The findings revealed clear positive associations between CMI and increased mortality related to all causes, premature, and cancer within the cohort. These associations remained significant even in the fully adjusted model. Subgroup and sensitivity analyses demonstrated that the associations generally remained stable. We further explored the potential mediating effect of inflammation-related indicators within these associations and found that the associations appeared to be partially mediated by inflammatory pathways. Therefore, monitoring CMI values in the overweight and obese adults offers a straightforward and accessible strategy for long-term health management.

The CMI, a relatively new anthropometric indicator, was first introduced in 2015 as a predictor of DM risk (8). Subsequent studies further investigated the relationship between CMI and DM in populations from different countries, and demonstrating an independent correlation between CMI and DM among Chinese, Japanese and US adults (9–11). Moreover, extensive investigations have demonstrated the significant positive association between CMI and adverse health outcomes, such as hypertension, CVD, and MetS (12–15, 23, 24). These findings underscore the close correlation of CMI with various systemic diseases, particularly those linked to adverse health outcomes of obesity. Nonetheless, there remains a lack of previous studies focusing on the associations of CMI with all-cause and cause-specific mortality among overweight and obese adults.

It is widely recognized that both abdominal obesity and lipid metabolism disorders are pivotal factors not only for DM, CVD, MetS, osteoporosis, and infertility, but also significant risk factors for all-cause, DM, CVD, and cancer mortality (25–28). Consequently, the CMI, which integrates parameters of abdominal obesity and blood lipid parameters, is considered an appropriate indicator for assessing metabolically unhealthy obesity and predicting mortality. Zakerkish et al. confirmed that metabolically unhealthy individuals, regardless of obesity status, exhibited significantly higher CMI values and an increased risk of cardiovascular disease (29). Similarly, Sun et al. established that elevated CMI values are associated with an increased risk of biological aging (30). With its growing clinical application, several studies have highlighted the prognostic value of CMI for all-cause, CVD, and cancer-related mortality among elderly and general populations (16–18). Xu et al. found a positive relationship between CMI and all-cause mortality (fully adjusted model: HR = 1.11, 95% CI: 1.01–1.21) among U.S. participants aged 65 years and older (16). Wang et al. reported no correlation between CMI and all-cause, DM, CVD, and cancer mortality in fully adjusted models, but a stronger positive association was observed between CMI and all-cause mortality (fully adjusted model: HR = 1.01, 95% CI: 1.01–1.11) among individuals aged <60 years (17). Liu et al. demonstrated a positive relationship between CMI and cancer mortality (fully adjusted model: HR = 1.05, 95% CI: 1.01–1.10), while also noting a negative correlation with all-cause mortality (fully adjusted model: baseline CMI <0.98, HR = 0.59, 95% CI: 0.43–0.82) within the general population (18).

Furthermore, the inverse L-shaped association was found between CMI, and risk of all-cause and cancer mortality. The possible mechanism may explain as “obesity paradox”. The obesity paradox, first described over 20 years ago in cardio-metabolic disease, is a medical hypothesis that suggests being overweight may provide a survival advantage in various illnesses (31). Recent studies have shown that cancer patients with low-normal BMI (or those with weight loss) have worse outcomes than obese patients (32). These results suggest that obesity has a protective effect and has been termed the “obesity paradox”. As demonstrated in Supplementary Table 1, the CMI values showed a clear increase with higher obesity class. However, the highest mortality rates were observed in the class II obesity group (35.0–39.9 kg/m^2^). There was no significant difference in all-cause and cancer mortality between the various classes of obesity. Nevertheless, our study found that CMI remained a positive correlation with mortality.

Although CMI is strongly associated with all-cause, premature, and cancer mortality, as shown by our study, the underlying biological mechanisms driving these associations remain unclear. Chronic low-grade systemic inflammation plays an important role in the onset and progression of obesity (3). Earlier research demonstrated that excessive body fat accumulation could induce an imbalanced production of various adipokines and promote the infiltration of macrophages and other immune cells in AT (4, 5). Elevated inflammatory markers are independently associated with increased tissue damage and mortality risks in obese adults, irrespective of other established risk factors (6, 7). Numerous studies have shown that components of the CBC and CBC-derived inflammatory indicators, tend to be elevated in individuals with obesity (33–36). These CBC and CBC-derived inflammatory indicators are also associated with increased mortality (37, 38). In this study, we found that CMI was positively associated with lymphocytes, neutrophils, monocytes, platelets, and inflammatory indexes (SII, AISI, and SIRI). Most inflammatory indicators were positively related to mortality. Consequently, the mediating roles of these inflammatory indicators were assessed, revealing that neutrophils mediated 16.27% of the correlation between CMI and all-cause mortality, and 11.01% of the association between CMI and premature mortality. The accumulation of fat in AT induces stress and dysfunction in adipocytes, triggering an inflammatory response. This is followed by the infiltration of AT by cells from the innate immune system (39, 40). Recent evidence indicates that neutrophils are the initial immune cells to infiltrate adipose tissue (39, 40). Once activated, these neutrophils release inflammatory factors that recruit macrophages and other immune cells (39, 40). In turn, these cells sustain the inflammatory state by producing cytokines and chemokines, which can spread to other parts of the body, leading to a systemic inflammatory condition (39–41). With persistent systemic inflammation, neutrophils are consistently recruited to the inflammation site, potentially exacerbating tissue damage in non-communicable autoinflammatory conditions (42). Recent studies have elucidated the mechanisms through which neutrophils undergo various forms of regulated cell death (42). Moreover, neutrophils are intrinsically linked to various health concerns, including CVD, cancer, and infections, by either protecting against, initiating, or exacerbating their effects on the host (43). Our findings confirm the significant mediating role of inflammation, particularly through neutrophils, providing validated evidence of their involvement in this association. These findings suggest that monitoring CMI values in adults with overweight or obesity provides a straightforward and practical method for effective long-term health management.

The present study boasts several strengths. A primary advantage is the use of data from NHANES and NCHS, part of a large-scale prospective cohort study, enhancing the representativeness of the findings. Furthermore, this study identified a positive association between the CMI and mortality related to all-cause, premature, and cancer among the overweight and obese adults. These findings suggest that CMI could serve as a valuable tool for early risk stratification and the formulation of intervention strategies within these populations. However, the present study still had several limitations. Firstly, it was conducted among U.S. adults with a relatively limited sample size, which may restrict the generalizability of the results to overweight and obese populations in different populations. Secondly, due to inherent study design constraints, it may not be possible to completely rule out all confounding factors. Thirdly, information on smoking and drinking behaviors, as well as histories of DM, hypertension, and hyperlipidemia, was collected through self-report questionnaires, making recall bias unavoidable.

## Conclusion

In the present study, we presented evidence supporting the positive associations of the CMI with increased mortality from all-cause, premature, and cancer among the overweight and obese U.S. adults. The associations appeared to be partially mediated by inflammatory pathways, suggesting a mechanism linking CMI to adverse health outcomes. Given that CMI is a relatively easy and effective metabolism-related indicator to measure, these findings may offer valuable insights for early risk stratification and the formulation of intervention strategies within overweight and obese populations.

## Data Availability

The original contributions presented in the study are included in the article/Supplementary Material, further inquiries can be directed to the corresponding author.

## Supplementary material

Supplementary Figure 1 RCS regression analysis for CMI with **(A)** all-cause, **(B)** premature, and **(C)** cancer mortality after imputation. (A1, B1, C1) Imputation 1; (A2, B2, C2) Imputation 2; (A3, B3, C3) Imputation 3; (A4, B4, C4) Imputation 4; (A5, B5, C5) Imputation 5. Adjust for age, gender, race, marital status, education level, PIR, smoking status, drinking status, DM, hypertension and hyperlipidemia. RCS, restricted cubic spline; CMI, cardiometabolic index; PIR: family poverty-to-income ratio; DM, diabetes mellitus.

## References

[1] NCD Risk Factor Collaboration (NCD-RisC). Worldwide trends in underweight and obesity from 1990 to 2022: a pooled analysis of 3663 population-representative studies with 222 million children, adolescents, and adults. Lancet. (2024) 403(10431):1027–50. 10.1016/S0140-6736(23)02750-2 38432237 PMC7615769

[2] Blüher M. Obesity: global epidemiology and pathogenesis. Nat Rev Endocrinol. (2019) 15:288–98. 10.1038/s41574-019-0176-8 30814686

[3] Cifuentes M Verdejo HE Castro PF Corvalan AH Ferreccio C Quest AFG Low-grade chronic inflammation: a shared mechanism for chronic diseases. Physiology (Bethesda). (2025) 40(1):4–25. 10.1152/physiol.00021.2024 39078396

[4] Suren Garg S Kushwaha K Dubey R Gupta J. Association between obesity, inflammation and insulin resistance: insights into signaling pathways and therapeutic interventions. Diabetes Res Clin Pract. (2023) 200:110691. 10.1016/j.diabres.2023.110691 37150407

[5] Uranga RM Keller JN. The complex interactions between obesity, metabolism and the brain. Front Neurosci. (2019) 13:513. 10.3389/fnins.2019.00513 31178685 PMC6542999

[6] Dibaba DT Judd SE Gilchrist SC Cushman M Pisu M Safford M Association between obesity and biomarkers of inflammation and metabolism with cancer mortality in a prospective cohort study. Metab Clin Exp. (2019) 94:69–76. 10.1016/j.metabol.2019.01.007 30802456 PMC7401298

[7] Kolb H. Obese visceral fat tissue inflammation: from protective to detrimental? BMC Med. (2022) 20(1):494. 10.1186/s12916-022-02672-y 36575472 PMC9795790

[8] Wakabayashi I Daimon T. The “cardiometabolic index” as a new marker determined by adiposity and blood lipids for discrimination of diabetes mellitus. Clin Chim Acta. (2015) 438:274–8. 10.1016/j.cca.2014.08.042 25199852

[9] Zha F Cao C Hong M Hou H Zhang Q Tang B The nonlinear correlation between the cardiometabolic index and the risk of diabetes: a retrospective Japanese cohort study. Front Endocrinol (Lausanne). (2023) 14:1120277. 10.3389/fendo.2023.1120277 36875460 PMC9980900

[10] Song J Li Y Zhu J Liang J Xue S Zhu Z. Non-linear associations of cardiometabolic index with insulin resistance, impaired fasting glucose, and type 2 diabetes among US adults: a cross-sectional study. Front Endocrinol (Lausanne). (2024) 15:1341828. 10.3389/fendo.2024.1341828 38410697 PMC10894973

[11] Liu Y Jiang H Luo L Gao Z. Relationship between four visceral obesity indices and prediabetes and diabetes: a cross-sectional study in Dalian, China. BMC Endocr Disord. (2024) 24(1):191. 10.1186/s12902-024-01718-x 39294627 PMC11409536

[12] Zou J Xiong H Zhang H Hu C Lu S Zou Y. Association between the cardiometabolic index and non-alcoholic fatty liver disease: insights from a general population. BMC Gastroenterol. (2022) 22(1):20. 10.1186/s12876-022-02099-y 35021995 PMC8756663

[13] Luo X Cai B. Association between cardiometabolic index and congestive heart failure among US adults: a cross-sectional study. Front Cardiovasc Med. (2024) 11:1433950. 10.3389/fcvm.2024.1433950 39318833 PMC11419996

[14] Lazzer S D'Alleva M Isola M De Martino M Caroli D Bondesan A Cardiometabolic index (CMI) and visceral adiposity index (VAI) highlight a higher risk of metabolic syndrome in women with severe obesity. J Clin Med. (2023) 12(9):3055. 10.3390/jcm12093055 37176497 PMC10179486

[15] Zhuo L Lai M Wan L Zhang X Chen R. Cardiometabolic index and the risk of new-onset chronic diseases: results of a national prospective longitudinal study. Front Endocrinol (Lausanne). (2024) 15:1446276. 10.3389/fendo.2024.144626 39497804 PMC11532088

[16] Xu B Wu Q La R Lu L Abdu FA Yin G Is systemic inflammation a missing link between cardiometabolic index with mortality? Evidence from a large population-based study. Cardiovasc Diabetol. (2024) 23(1):212. 10.1186/s12933-024-02251-w 38902748 PMC11191290

[17] Wang J Xiao L Li Z. Cardiometabolic index and mortality risks: elevated cancer and reduced cardiovascular mortality risk in a large cohort. Lipids Health Dis. (2024) 23(1):427. 10.1186/s12944-024-02415-3 39736689 PMC11687173

[18] Liu M Wang C Liu R Wang Y Wei B. Association between cardiometabolic index and all-cause and cause-specific mortality among the general population: NHANES 1999–2018. Lipids Health Dis. (2024) 23(1):425. 10.1186/s12944-024-02408-2 39731068 PMC11681656

[19] Bundy JD Mills KT He H LaVeist TA Ferdinand KC Chen J Social determinants of health and premature death among adults in the USA from 1999 to 2018: a national cohort study. Lancet Public Health. (2023) 8:e422–431. 10.1016/S2468-2667(23)00081-6 37244672 PMC10349537

[20] Xiu J Lin X Chen Q Yu P Lu J Yang Y The aggregate index of systemic inflammation (AISI): a novel predictor for hypertension. Front Cardiovasc Med. (2023) 10:1163900. 10.3389/fcvm.2023.1163900 37265570 PMC10229810

[21] Wang RH Wen WX Jiang ZP Du ZP Ma ZH Lu AL The clinical value of neutrophil-to-lymphocyte ratio (NLR), systemic immune-inflammation index (SII), platelet-to-lymphocyte ratio (PLR) and systemic inflammation response index (SIRI) for predicting the occurrence and severity of pneumonia in patients with intracerebral hemorrhage. Front Immunol. (2023) 14:1115031. 10.3389/fimmu.2023.1115031 36860868 PMC9969881

[22] Wang G Fang L Chen Y Ma Y Zhao H Wu Y Association between exposure to mixture of heavy metals and hyperlipidemia risk among U.S. adults: a cross-sectional study. Chemosphere. (2023) 344:140334. 10.1016/j.chemosphere.2023.140334 37788750

[23] Huang X Wen S Huang Y Zhang B Xia Z Huang Z. Association between cardiometabolic index and the incidence of stroke: a prospective nationwide cohort study in China. J Diabetes Metab Disord. (2024) 24(1):26. 10.1007/s40200-024-01530-3 39735172 PMC11680538

[24] Datta Banik S Pacheco-Pantoja E Lugo R Gómez-de-Regil L Chim Aké R Méndez González RM Evaluation of anthropometric indices and lipid parameters to predict metabolic syndrome among adults in Mexico. Diabetes Metab Syndr Obes. (2021) 14:691–701. 10.2147/DMSO.S281894 33623404 PMC7896767

[25] Wilcox NS Amit U Reibel JB Berlin E Howell K Ky B. Cardiovascular disease and cancer: shared risk factors and mechanisms. Nat Rev Cardiol. (2024) 21(9):617–31. 10.1038/s41569-024-01017-x 38600368 PMC11324377

[26] Bhaskaran K Dos-Santos-Silva I Leon DA Douglas IJ Smeeth L. Association of BMI with overall and cause-specific mortality: a population-based cohort study of 3·6 million adults in the UK. Lancet Diabetes Endocrinol. (2018) 6(12):944–53. 10.1016/S2213-8587(18)30288-2 30389323 PMC6249991

[27] Dai D Chen B Wang B Tang H Li X Zhao Z Pretreatment TG/HDL-C ratio is superior to triacylglycerol level as an independent prognostic factor for the survival of triple negative breast cancer patients. J Cancer. (2016) 7(12):1747–54. 10.7150/jca.15776 27698913 PMC5039397

[28] Zhou Z Liu Q Zheng M Zuo Z Zhang G Shi R Comparative study on the predictive value of TG/HDL-C, TyG and TyG-BMI indices for 5-year mortality in critically ill patients with chronic heart failure: a retrospective study. Cardiovasc Diabetol. (2024) 23(1):213. 10.1186/s12933-024-02308-w 38902757 PMC11191322

[29] Zakerkish M Hoseinian A Alipour M Payami SP. The association between cardio-metabolic and hepatic indices and anthropometric measures with metabolically obesity phenotypes: a cross-sectional study from the Hoveyzeh Cohort Study. BMC Endocr Disord. (2023) 23(1):122. 10.1186/s12902-023-01372-9 37246210 PMC10226206

[30] Sun M Bao S. Association between cardiometabolic index and biological aging in the US population: evidence from NHANES 2015–2020. Front Aging Neurosci. (2024) 16:1507035. 10.3389/fnagi.2024.1507035 39679260 PMC11638210

[31] Tutor AW Lavie CJ Kachur S Milani RV Ventura HO. Updates on obesity and the obesity paradox in cardiovascular diseases. Prog Cardiovasc Dis. (2023) 78:2–10. 10.1016/j.pcad.2022.11.013 36481212

[32] Lee DH Giovannucci EL. The obesity paradox in cancer: epidemiologic insights and perspectives. Curr Nutr Rep. (2019) 8(3):175–81. 10.1007/s13668-019-00280-6 31129887

[33] Çömlek FÖ Körez MK. Assessments of the relationship between inflammatory parameters from complete blood count and clinical findings in children with obesity and comparison with healthy children. Metab Syndr Relat Disord. (2025) 23(5):281–7. 10.1089/met.2025.0023 40256812

[34] Chen Y Huang R Mai Z Chen H Zhang J Zhao L Association between systemic immune-inflammatory index and diabetes mellitus: mediation analysis involving obesity indicators in the NHANES. Front Public Health. (2024) 11:1331159. 10.3389/fpubh.2023.1331159 38269383 PMC10806151

[35] Marra A Bondesan A Caroli D Sartorio A. Complete blood count (CBC)-derived inflammation indexes are useful in predicting metabolic syndrome in adults with severe obesity. J Clin Med. (2024) 13(5):1353. 10.3390/jcm13051353 38592161 PMC10932131

[36] Gomez-Casado G Jimenez-Gonzalez A Rodriguez-Muñoz A Tinahones FJ González-Mesa E Murri M Neutrophils as indicators of obesity-associated inflammation: a systematic review and meta-analysis. Obes Rev. (2025) 26(3):e13–868. 10.1111/obr.13868 PMC11791391 39610288

[37] Chan SHT Yu T Zhang Z Chang LY Guo C Bo Y Total and differential white blood cell count and cause-specific mortality in 436750 Taiwanese adults. Nutr Metab Cardiovasc Dis. (2022) 32(4):937–47. 10.1016/j.numecd.2021.11.004 35078679

[38] Dong G Gu X Qiu C Xie Y Hu Z Wu L. Neutrophil-lymphocyte ratio is a predictor for all-cause and cardiovascular mortality in individuals with prediabetes in a national study. Endocrine. (2025) 87(2):589–98. 10.1007/s12020-024-04075-w 39438396

[39] Uribe-Querol E Rosales C. Neutrophils actively contribute to obesity-associated inflammation and pathological complications. Cells. (2022) 11(12):1883. 10.3390/cells11121883 35741012 PMC9221045

[40] Altamura S Lombardi F Palumbo P Cinque B Ferri C Del Pinto R The evolving role of neutrophils and neutrophil extracellular traps (NETs) in obesity and related diseases: recent insights and advances. Int J Mol Sci. (2024) 25(24):13633. 10.3390/ijms252413633 39769394 PMC11727698

[41] Liew PX Kubes P. The neutrophil’s role during health and disease. Physiol Rev. (2019) 99(2):1223–48. 10.1152/physrev.00012.2018 30758246

[42] Dejas L Santoni K Meunier E Lamkanfi M. Regulated cell death in neutrophils: from apoptosis to NETosis and pyroptosis. Semin Immunol. (2023) 70:101849. 10.1016/j.smim.2023.101849 37939552 PMC10753288

[43] Aroca-Crevillén A Vicanolo T Ovadia S Hidalgo A. Neutrophils in physiology and pathology. Annu Rev Pathol. (2024) 19:227–59. 10.1146/annurev-pathmechdis-051222-015009 38265879 PMC11060889

