# Supplementary material for: Association of cardiometabolic index with all-cause and cause-specific mortality among overweight and obese adults: a cohort study
Source: Front Cardiovasc Med. 2025 Jun 19;12:1610257. doi: 10.3389/fcvm.2025.1610257 (PMC12222087; doi:10.3389/fcvm.2025.1610257)
Supplement: Supplementary file 1 [file Datasheet1.pdf]

Supplementary Table 1. The CMI, inflammation, and mortality data in different obesity class groups

	Overweight<br>25.0-29.9 kg/m^2	Class I obesity<br>30.0-34.9 kg/m^2	Class II obesity<br>35.0-39.9 kg/m^2	Class III obesity<br>≥ 40.0 kg/m^2		
N[a]	6524	4006	1793	1351		
CMI, Mean (SE)	0.73 (0.01)	0.92 (0.01)	1.05 (0.02)	1.14 (0.03)	F=99.32	<0.001
Lymphocytes, 1000 cells/uL, Mean (SE)	1.97 (0.01)	2.06 (0.01)	2.14 (0.02)	2.21 (0.02)	F=42.82	<0.001
Neutrophils, 1000 cell/uL, Mean (SE)	3.89 (0.03)	4.10 (0.03)	4.40 (0.05)	4.70 (0.07)	F=67.67	<0.001
Monocytes, 1000 cells/uL, Mean (SE)	0.54 (0.00)	0.55 (0.00)	0.55 (0.01)	0.57 (0.01)	F=7.51	<0.001
Platelets, 1000 cells/uL, Mean (SE)	(1.19)	256.70 (1.59)	263.73 (2.39)	273.17 (3.20)	F=22.41	<0.001
SII, Mean (SE)	534.57 (5.54)	548.25 (6.66)	587.65 (11.36)	621.00 (13.20)	F=17.02	<0.001
AISI, Mean (SE)	295.67 (3.96)	309.56 (5.36)	334.60 (8.55)	357.69 (8.86)	F=18.10	<0.001
SIRI, Mean (SE)	1.17 (0.01)	1.20 (0.02)	1.25 (0.03)	1.32 (0.03)	F=8.97	<0.001
All-cause mortality, yes, N (%)	603 (8.29)	413 (9.81)	188 (10.67)	136 (9.55)	χ^2=13.07	0.058
Premature mortality, yes, N (%)	376 (5.29)	251 (6.51)	109 (7.01)	103 (7.59)	χ^2=16.24	0.027
Cancer mortality, yes, N (%)	166 (2.42)	115 (2.91)	55 (2.45)	28 (1.99)	χ^2=4.20	0.547
DM mortality, yes, N (%)	20 (0.17)	20 (0.29)	13 (0.63)	17 (1.05)	χ^2=28.23	<0.001
Cardiovascular mortality, yes, N (%)	342 (2.33)	140 (1.85)	109 (2.60)	51 (3.24)	χ^2=15.88	0.028
Follow-up time, months, Mean (SE)	139.94 (1.62)	131.04 (2.34)	127.89 (2.65)	119.52 (3.70)	F=14.08	<0.001

Mean (SE) for continuous variables. N (%) for categorical variables. N, number SE, standard error; CMI, cardiometabolic index; SII, systemic immune-inflammation index; AISI, aggregate index of systemic inflammation; SIRI, systemic inflammation response index.

[a] Unweighted number of observations in dataset.

Supplementary Table 2. The associations of BMI, WHtR and TG/HDL-c with mortality.

	Model 1		Model 2		Model 3	
Characteristic	HR (95%CI)	*P*	HR (95%CI)	*P*	HR (95%CI)	*P*
All-cause mortality						
BMI	1.02 (1.01 - 1.03)	<0.001	1.02 (1.01, 1.04)	0.002	1.01 (1.00, 1.03)	0.107
WHtR	85.39 (42.79, 170.37)	<0.001	13.68 (4.82, 38.87)	<0.001	6.39 (2.15, 18.99)	<0.001
TG/HDL-c	1.18 (1.10, 1.27)	<0.001	1.10 (1.02, 1.18)	0.018	1.07 (0.99, 1.15)	0.094
Premature mortality						
BMI	1.03 (1.02, 1.04)	<0.001	1.03 (1.02, 1.05)	<0.001	1.01 (1.00, 1.03)	0.112
WHtR	78.61 (33.81, 182.75)	<0.001	27.79 (8.07, 95.72)	<0.001	7.71 (1.99, 29.95)	0.003
TG/HDL-c	1.26 (1.16, 1.36)	<0.001	1.16 (1.07, 1.27)	<0.001	1.12 (1.03, 1.23)	0.012
Cancer mortality						
BMI	1.00 (0.98, 1.02)	0.950	1.00 (0.98, 1.02)	0.969	1.00 (0.97, 1.02)	0.799
WHtR	28.09 (8.25, 95.68)	<0.001	2.43 (0.41, 14.36)	0.327	1.76 (0.27, 11.30)	0.554
TG/HDL-c	1.22 (1.09, 1.37)	<0.001	1.18 (1.04, 1.34)	0.013	1.19 (1.05, 1.35)	0.006

Model 1: no covariates were adjusted. Model 2: age, gender, race, marital status, education level, PIR, smoking status, and drinking status. Model 3: age, gender, race, marital status, education level, PIR, smoking status, drinking status, DM, hypertension, and hyperlipidemia were adjusted. PIR: family poverty-to-income ratio; BMI, body mass index; WHtR, waist to height ratio; TG, triglyceride; DM, diabetes mellitus.

Supplementary Table 3. Subgroup analysis and interaction analysis of CMI on mortality.

Variables	All-cause mortality			Premature mortality			Cancer mortality		
	HR (95% CI)	*P*	*P* for interaction	HR (95% CI)	*P*	*P* for interaction	HR (95% CI)	*P*	*P* for interaction
Gender			0.859			0.149			0.649
Male	1.15 (0.99, 1.32)	0.062		1.19 (1.00, 1.42)	0.055		1.32 (0.99, 1.74)	0.054	
Female	1.14 (0.91, 1.42)	0.258		1.31 (1.05, 1.64)	0.017		1.37 (1.01, 1.85)	0.043	
Age			0.020			0.405			0.393
< 65	1.22 (1.03, 1.43)	0.019		1.25 (1.06, 1.48)	0.008		1.34 (1.02, 1.77)	0.036	
≥ 65	1.05 (0.89, 1.25)	0.558		1.05 (0.79, 1.39)	0.749		1.26 (0.94, 1.69)	0.127	
BMI			0.938			0.737			0.693
< 30	1.15 (0.97, 1.36)	0.098		1.18 (0.96, 1.46)	0.120		1.29 (0.93, 1.79)	0.130	
≥ 30	1.13 (0.93, 1.38)	0.228		1.29 (1.06, 1.58)	0.013		1.39 (1.08, 1.78)	0.010	
Race/ethnicity			0.002			0.017			0.163
Mexican American	1.03 (0.83, 1.29)	0.760		1.01 (0.74, 1.37)	0.960		0.88 (0.57, 1.38)	0.586	
Other Hispanic	2.62 (1.71, 4.02)	<0.001		2.81 (1.65, 4.78)	<0.001		2.04 (1.09, 3.84)	0.027	
Non-Hispanic White	1.06 (0.92, 1.22)	0.441		1.17 (0.99, 1.38)	0.066		1.30 (1.04, 1.63)	0.022	
Non-Hispanic Black	1.72 (1.30, 2.28)	<0.001		1.80 (1.38, 2.36)	<0.001		2.05 (1.21, 3.45)	0.007	
Other Race	0.81 (0.46, 1.42)	0.462		0.92 (0.50, 1.70)	0.786		0.81 (0.26, 2.53)	0.710	
Education level			0.056			0.088			0.065
Less Than 9th Grade	1.06 (0.80, 1.42)	0.672		1.11 (0.76, 1.62)	0.598		0.85 (0.55, 1.32)	0.467	
9-11th Grade	1.20 (0.97, 1.48)	0.087		1.26 (1.02, 1.56)	0.030		1.84 (1.22, 2.78)	0.004	
High School or Equivalent	1.16 (0.90, 1.50)	0.249		1.17 (0.88, 1.56)	0.287		1.40 (0.98, 2.01)	0.066	
Some College or AA degree	1.38 (1.09, 1.76)	0.008		1.50 (1.11, 2.02)	0.008		1.59 (1.16, 2.17)	0.004	
College Graduate or above	0.84 (0.61, 1.15)	0.268		1.14 (0.84, 1.56)	0.394		0.86 (0.53, 1.40)	0.547	
Marital status			0.012			0.020			0.481
Married	1.13 (0.97, 1.32)	0.108		1.23 (1.02, 1.49)	0.029		1.28 (0.96, 1.70)	0.092	
Widowed	0.91 (0.56, 1.49)	0.706		1.50 (0.90, 2.49)	0.117		1.42 (0.72, 2.79)	0.307	
Divorced	1.06 (0.81, 1.39)	0.688		1.06 (0.77, 1.44)	0.726		1.19 (0.81, 1.76)	0.381	

Separated	5.36 (2.71, 10.59)	<0.001		6.58 (2.85, 15.21)	<0.001		4.63 (1.28, 16.81)	0.020	
Never married	1.33 (0.91, 1.96)	0.140		1.36 (0.92, 2.00)	0.120		1.87 (1.13, 3.10)	0.015	
Living with partner	0.75 (0.44, 1.28)	0.295		0.83 (0.50, 1.38)	0.478		0.57 (0.07, 4.51)	0.592	
Smoking status			0.042			0.018			0.963
Yes	1.07 (0.93, 1.25)	0.345		1.13 (0.95, 1.34)	0.167		1.34 (1.01, 1.77)	0.041	
No	1.39 (1.16, 1.66)	<0.001		1.69 (1.36, 2.10)	<0.001		1.37 (1.01, 1.86)	0.042	
Drinking status			0.443			0.089			0.229
Yes	1.14 (1.00, 1.30)	0.043		1.13 (0.96, 1.33)	0.128		1.43 (1.14, 1.80)	0.002	
No	1.13 (0.92, 1.39)	0.257		1.64 (1.30, 2.07)	<0.001		1.16 (0.80, 1.68)	0.426	
DM			0.775			0.645			0.642
Yes	1.16 (0.98, 1.36)	0.081		1.24 (1.03, 1.48)	0.022		1.25 (0.86, 1.82)	0.239	
No	1.12 (0.92, 1.36)	0.247		1.22 (0.99, 1.52)	0.066		1.38 (1.06, 1.78)	0.015	
Hypertension			0.258			0.627			0.048
Yes	1.08 (0.92, 1.26)	0.339		1.21 (1.01, 1.45)	0.042		1.06 (0.79, 1.41)	0.697	
No	1.28 (1.03, 1.60)	0.026		1.28 (0.99, 1.65)	0.062		1.83 (1.40, 2.39)	<0.001	
Hyperlipidemia			0.022			0.092			0.467
Yes	1.12 (0.99, 1.28)	0.075		1.23 (1.06, 1.42)	0.006		1.34 (1.09, 1.65)	0.006	
No	3.69 (1.37, 9.96)	0.010		2.74 (0.94, 7.96)	0.065		1.29 (0.27, 6.13)	0.745	

Adjusted for age, gender, race, marital status, education level, PIR, smoking status, drinking status, DM, hypertension, and hyperlipidemia history. The model is not adjusted for the stratification variable. HR, hazard ratio; 95% CI, 95% confidence interval; CMI, cardiometabolic index; PIR, family poverty-to-income ratio; BMI, body mass index; DM, diabetes mellitus.

Supplementary Table 4. Analysis of the mediation by inflammation-related indicators of the associations of CMI with mortality.

	Total effect (95%CI)	*P*	Direct effect (95%CI)	*P*	Indirect effect (95%CI)	*P*	Mediation, %
All-cause mortality							
Neutrophils	122.34 (46.68, 208.52)	<0.001	103.60 (32.60, 188.39)	<0.001	18.74 (11.29, 25.96)	<0.001	16.27
Premature mortality							
Neutrophils	165.80 (26.06, 282.13)	0.040	147.12 (11.40, 265.36)	0.040	18.68 (7.28, 30.74)	<0.001	11.01
Monocytes	171.50 (29.17, 288.03)	<0.001	163.73 (21.43, 280.18)	<0.001	7.77 (1.82, 14.48)	<0.001	4.27
SII	165.75 (23.89, 280.58)	0.040	162.60 (20.98, 277.49)	0.040	3.15 (0.80, 6.14)	<0.001	1.89
AISI	167.56 (25.90, 281.93,)	0.040	162.58 (21.54, 276.35)	0.040	4.98 (2.01, 8.17)	<0.001	2.98
SIRI	169.46 (26.89, 290.41)	0.040	162.68 (19.96, 285.85)	0.040	6.79 (0.56, 13.19)	<0.001	4.31
Cancer mortality							
SII	6.06 (5.94, 6.21)	<0.001	5.90 (5.89, 5.91)	<0.001	0.16 (0.04, 0.31)	<0.001	2.60
AISI	6.06 (5.99, 6.14)	<0.001	5.94 (5.94, 5.94)	<0.001	0.12 (0.05, 0.21)	<0.001	2.07

Adjust for age, gender, race, marital status, education level, PIR, smoking status, drinking status, DM, hypertension, and hyperlipidemia. SII, systemic immune-inflammation index; AISI, aggregate index of systemic inflammation; SIRI, systemic inflammation response index; PIR: family poverty-to-income ratio; DM, diabetes mellitus.
