# Supplementary figures and images for: Association of cardiometabolic index with all-cause and cause-specific mortality among overweight and obese adults: a cohort study
Source: Front Cardiovasc Med. 2025 Jun 19;12:1610257. doi: 10.3389/fcvm.2025.1610257 (PMC12222087; doi:10.3389/fcvm.2025.1610257)

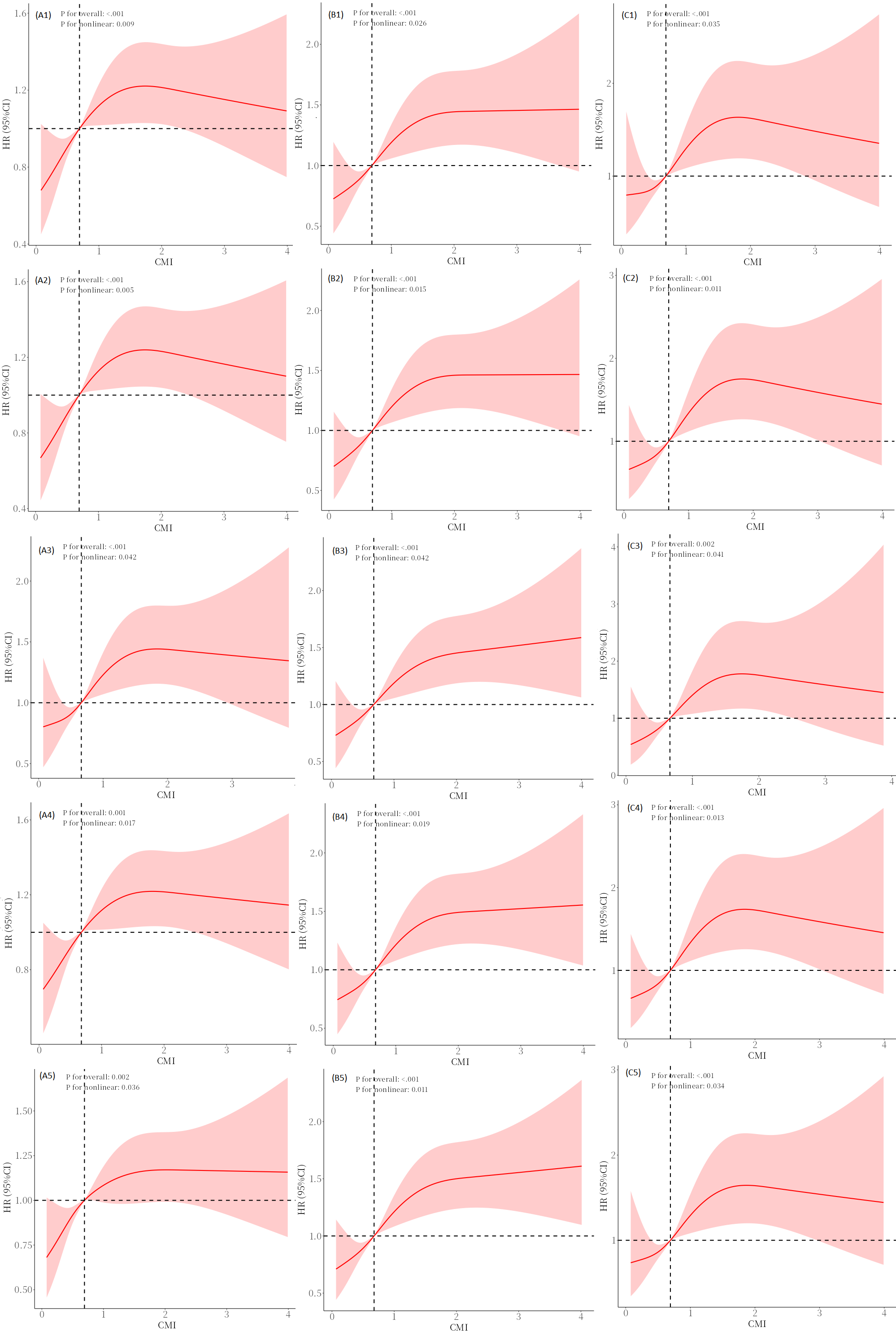

Supplement: Supplementary Figure 1 — RCS regression analysis for CMI with (A) all-cause, (B) premature, and (C) cancer mortality after imputation. (A1, B1, C1) Imputation 1; (A2, B2, C2) Imputation 2; (A3, B3, C3) Imputation 3; (A4, B4, C4) Imputation 4; (A5, B5, C5) Imputation 5. Adjust for age, gender, race, marital status, education level, PIR, smoking status, drinking status, DM, hypertension and hyperlipidemia. RCS, restricted cubic spline; CMI, cardiometabolic index; PIR: family poverty-to-income ratio; DM, diabetes mellitus. [file Image1.tif]
